# An investigation of the relationship between grit, physical activity, and self-efficacy: a variable-centered and person-centered approach

**DOI:** 10.3389/fpsyg.2025.1742211

**Published:** 2026-01-06

**Authors:** Bin Chen, Shushu Hua, Zaibin Tu, Chang Hu, Jing Yang

**Affiliations:** 1 Physical Education College, Jiangxi Normal University, Nanchang, China; 2 College of Humanities and Social Sciences, Zhongkai University of Agriculture and Engineering, Guangzhou, China; 3 School of Educational Science, Hanshan Normal University, Chaozhou, China; 4 School of Psychology and Sociology, Mianyang Normal University, Mianyang, China

**Keywords:** grit, latent profile analysis, mediation analysis, physical activity, self-efficacy, university students

## Abstract

**Background:**

Grit, defined as perseverance and passion for long-term goals, is a vital psychological trait that contributes to academic success and overall wellbeing. At the same time, regular PA supports physical and mental health, yet its engagement often declines among university students. Although grit has been linked to health-promoting behaviors, the mechanisms underlying this association remain unclear. This study investigated the mediating role of self-efficacy from a variable-centered perspective and identified student profiles based on grit and self-efficacy through a person-centered approach.

**Methods:**

A cross-sectional survey of 3,752 Chinese university students was conducted. Structural equation modeling tested the mediating role of self-efficacy in the grit-PA relationship. Latent Profile Analysis identified subgroups with distinct combinations of grit and self-efficacy, with PA levels compared across profiles.

**Results:**

The variable-centered analysis revealed that grit was positively associated with PA (*β* = 0.370, *p* < 0.001), with self-efficacy serving as a significant partial mediator, accounting for 21% of the total effect (indirect effect = 0.077, 95% CI [0.058, 0.097]). The person-centered analysis identified three distinct profiles: “Limited Self-Regulation” (16.12%), “Moderate Self-Regulation” (55.84%), and “Strong Self-Regulation” (28.04%). Students in the Strong Self-Regulation profile demonstrated significantly higher PA engagement (*M* = 49.03) compared to those in the Moderate (*M* = 31.56) and Limited (*M* = 17.84) profiles (*F* = 225.11, *p* < 0.001, η^2^ = 0.107).

**Conclusion:**

Self-efficacy is consistent with a mediating role linking grit to PA among university students. The identification of three distinct profiles reveals meaningful heterogeneity in motivational configurations. Students with high grit and self-efficacy demonstrate optimal PA engagement, while those low in both represent a vulnerable subgroup requiring targeted intervention. Integrating variable- and person-centered approaches provides a comprehensive understanding of motivational processes underlying active lifestyles in higher education, emphasizing the need for tailored, profile-specific interventions.

## 1 Introduction

Physical inactivity is now recognized as a significant global public health challenge (Santos et al., 2023; van Sluijs et al., 2021; Dogra et al., 2022). According to the World Health Organisation, approximately 31% of adults fail to meet even the minimum recommended levels of physical activity (PA), a shortfall that directly contributes to the rising prevalence of chronic diseases (Guthold et al., 2020; Strain et al., 2024; Morouço et al., 2022; Chen et al., 2025). University students sit at a pivotal developmental juncture: they are transitioning into adulthood while simultaneously laying the foundations of their future professional and personal lives (Maggs, 2024). Their health behaviors, therefore, have consequences that reverberate far beyond the campus, shaping labor-force productivity, healthcare demand, and ultimately societal wellbeing (Sanci et al., 2022; Almoraie et al., 2025; Jong et al., 2025). However, accumulating evidence suggests that students’ physical activity levels decline sharply after they enter higher education (Hammer et al., 2025; Deforche et al., 2015). Confronted with heavy academic workloads, social pressures, and career preparation, many struggle to maintain regular exercise routines (Hilger-Kolb et al., 2020; Brown et al., 2024). The resulting sedentariness heightens their risk for cardiometabolic conditions and is linked to increased anxiety, depression, and stress (Booth et al., 2023; Biddle et al., 2023; Biddle et al., 2021).

Most investigations into student inactivity have focused on external barriers, such as crowded timetables, limited facilities, high membership fees, or a lack of social support (Griffiths et al., 2022; Moore et al., 2023; Silva et al., 2022). Although valuable, such work cannot fully explain why two students exposed to the same campus environment often display markedly different exercise patterns. This discrepancy suggests that intrapersonal factors—particularly enduring psychological attributes—may be decisive in determining who persists in PA when circumstances are unfavorable (Vella et al., 2023; Wilhite et al., 2023; Martín-Rodríguez et al., 2024). Accordingly, the present study shifts the analytic lens from external constraints to internal motivation, examining how positive psychological traits are associated with students’ exercise behavior.

Among the traits highlighted by contemporary positive psychology, grit—defined as sustained perseverance and passion for long-term goals (Duckworth et al., 2007)—has attracted substantial attention. Individuals high in grit show greater Resilience in the face of setbacks and a stronger willingness to invest effort over extended periods (Calo et al., 2024; Biggs et al., 2024; Sulla et al., 2022). Applied to health behavior, gritty students may endure the fatigue, time pressure, and monotony that often accompany training, viewing exercise as an indispensable pathway to long-term health (Hartzel et al., 2021; Bae et al., 2024; Estep et al., 2021). Despite the intuitive appeal of this link, the psychological mechanism translating grit into actual PA remains under-specified (Rutberg et al., 2020).

One plausible mediator is self-efficacy—the belief in one’s capability to organize and execute courses of action required to attain a goal (Bandura, 1997). Social Cognitive Theory posits that self-efficacy is a proximal determinant of both the initiation and maintenance of behavior (Schwarzer and Renner, 2000; Bandura, 1991). Students with higher grit are more likely to persist through repeated bouts of practice, thereby accumulating mastery experiences that fortify their confidence in exercising successfully (Daniels et al., 2021). Heightened exercise-specific self-efficacy, in turn, should enhance motivation, goal setting, and behavioral persistence (Blom et al., 2021; Martinez Kercher et al., 2024). Thus, self-efficacy may function as the cognitive “bridge” that channels grit into sustained PA (Dai et al., 2025).

However, traditional variable-centered analyses implicitly assume that all individuals follow the same psychological pathway, risking the oversight of meaningful heterogeneity (Liu et al., 2024; Stiller et al., 2023). In reality, the interplay between grit and self-efficacy may vary considerably. Some students may exhibit both persistence and confidence, while others may show perseverance without corresponding self-belief, or vice versa. To address these gaps, the present study integrates variable- and person-centered methodologies. We first test whether self-efficacy mediates the grit–PA relationship at the population level, and then employ latent profile analysis to uncover distinct psychological profiles and compare their physical activity engagement. The findings are expected to provide actionable insights for designing tailored, evidence-based interventions that promote active lifestyles in higher education settings.

## 2 Theoretical framework and hypotheses

### 2.1 Grit and physical activity

Grit is a key positive personality trait that reflects the ability to sustain effort and passion toward long-term, meaningful goals (Jiang et al., 2023). Duckworth et al. (2021) conceptualized grit as consisting of two dimensions: consistency of interest and perseverance of effort. In the context of PA, these dimensions jointly serve as an internal motivational foundation that supports continuous participation (Rosenkranz et al., 2023; Wang et al., 2021). Consistency of interest refers to maintaining long-term enthusiasm for health or fitness improvement rather than engaging in short-lived or impulsive exercise (Tross et al., 2024). Individuals with high consistency of interest can concentrate on their goals despite competing distractions or time pressures (Buabang et al., 2025). Perseverance of effort captures the ability to remain determined and psychologically resilient in the face of challenges such as muscle fatigue, time constraints, plateaus in progress, or minor injuries (Biggs et al., 2024).

Empirical evidence consistently supports a positive link between grit and PA (Cormier et al., 2024). Studies of college athletes have found that individuals with higher grit demonstrate greater training involvement and better performance outcomes (Moles et al., 2017). Research in general student populations also suggests that grit positively predicts both the frequency and duration of moderate-to-vigorous PA (Yu et al., 2024). This relationship may be explained through self-regulation theory, as gritty individuals exhibit stronger self-control and are more capable of postponing immediate gratification for long-term health benefits (Dorina et al., 2023). They tend to perceive discomfort or difficulty as a natural element of progress rather than as a reason to withdraw, which is linked to stronger adherence and persistence in exercise. The following hypothesis is thus proposed:

*Hypothesis 1 (H1)*: Grit is positively associated with university students’ PA levels.

### 2.2 The mediating role of self-efficacy

Although grit provides the motivational foundation for persistence, its influence on behavior operates through cognitive mechanisms (Jiang et al., 2023). Self-efficacy, as proposed in Bandura’s Social Cognitive Theory, represents the belief that one can effectively perform actions to achieve specific goals (Pajares, 1996). It is a primary determinant of behavior initiation and maintenance (Phillips and More, 2022; Hennessy et al., 2020).

The present study posits that self-efficacy mediates the relationship between grit and PA, operating through two interrelated processes. First, grit facilitates the development of self-efficacy (De La Cruz et al., 2021; Alhadabi and Karpinski, 2020). Bandura identified mastery experiences as the most influential source of efficacy (Welch and West, 1995). Students with high grit persist in their training efforts despite difficulties, which enables them to accumulate small but meaningful successes, such as improving their endurance or learning a new movement (Cocić et al., 2024). Each successful experience reinforces their confidence in their abilities and gradually strengthens their exercise-related self-efficacy (Wang et al., 2022). Prior research in educational and occupational settings has confirmed that grit is positively associated with self-efficacy (Zhou and Hou, 2025).

Second, self-efficacy acts as a direct motivator of behavior (Shengyao et al., 2024). Individuals with strong self-efficacy are more likely to initiate and maintain PA (Xie et al., 2025). They tend to set clear and challenging fitness goals, interpret fatigue or scheduling pressures as manageable, and recover faster from failure or frustration (Hull and Vultaggio, 2019). Substantial evidence from health psychology supports self-efficacy as a robust predictor of PA engagement and persistence (Chu et al., 2021; Maio et al., 2020).

Together, these two pathways suggest that grit enhances self-efficacy through mastery experiences, and heightened self-efficacy, in turn, promotes sustained participation in exercise. The following hypothesis is thus proposed:

*Hypothesis 2 (H2)*: Self-efficacy mediates the relationship between grit and PA.

### 2.3 A person-centered perspective on grit and self-efficacy

Traditional variable-centered approaches, such as regression or mediation analyses, typically assume structural homogeneity within the population (Woo et al., 2024). They focus on average relationships between variables, presuming that the exact underlying psychological mechanisms apply uniformly across individuals. However, this assumption often masks meaningful within-group variability and may overlook distinct subtypes of individuals who exhibit different psychological configurations (Huang et al., 2025b). To capture this underlying heterogeneity, a person-centered perspective provides a complementary analytic framework.

The LPA enables researchers to identify unobserved subgroups within a population based on shared patterns across multiple continuous variables (Beattie et al., 2022). In the context of this study, grit and self-efficacy are unlikely to have identical effects for all individuals. Instead, these traits may combine in diverse ways to form qualitatively distinct psychological profiles, each associated with unique patterns of exercise motivation and behavior. For example, some students may exhibit both high perseverance and strong self-belief, whereas others may be persistent but lack confidence, or confident but inconsistent in maintaining effort. These nuanced combinations cannot be adequately explained through variable-centered methods alone.

Theoretically, Self-Determination Theory (SDT) provides a valuable lens through which to understand this heterogeneity. SDT emphasizes that sustained behavioral engagement arises from the alignment of intrinsic motivation and perceived competence, a conceptually related concept to self-efficacy (Ryan and Deci, 2000). When individuals possess both a strong internal drive (reflecting grit) and a robust sense of competence (reflecting self-efficacy), they are more likely to internalize health-related goals, experience autonomous motivation, and maintain consistent PA (Dunston et al., 2020). Conversely, an imbalance between effort and confidence may be associated with motivational conflict and behavioral inconsistency.

Recent research employing person-centered approaches in positive psychology supports the value of this perspective. Studies have demonstrated that distinct psychological profiles based on constellations of positive traits, such as Resilience, optimism, and self-efficacy, are differentially associated with outcomes including academic achievement, emotional well-being, and adaptive coping (Sabouripour et al., 2021; Egan et al., 2021; Popa-Velea et al., 2021; Ye et al., 2024). Applying this framework to the present study, latent profile analysis is expected to reveal subgroups of university students characterized by distinct configurations of grit and self-efficacy. By examining how these profiles differ in their engagement in PA, the study aims to uncover the nuanced interplay between personal dispositions and health behavior, thereby extending current understanding beyond average effects. The following hypothesis is thus proposed:

*Hypothesis 3 (H3)*: Distinct latent profiles of university students can be identified based on their levels of grit and self-efficacy.

*Hypothesis 4 (H4)*: These latent profiles differ significantly in PA levels.

### 2.4 Aims of the study

The present study aims to (Santos et al., 2023) examine the mediating role of self-efficacy in the relationship between grit and PA among university students, and (van Sluijs et al., 2021) identify distinct psychological profiles combining grit and self-efficacy to explore group differences in PA engagement. These dual approaches aim to advance theoretical understanding and provide practical insights for designing targeted health promotion interventions in higher education contexts.

## 3 Materials and methods

### 3.1 Participants and procedure

**Figure 1:**
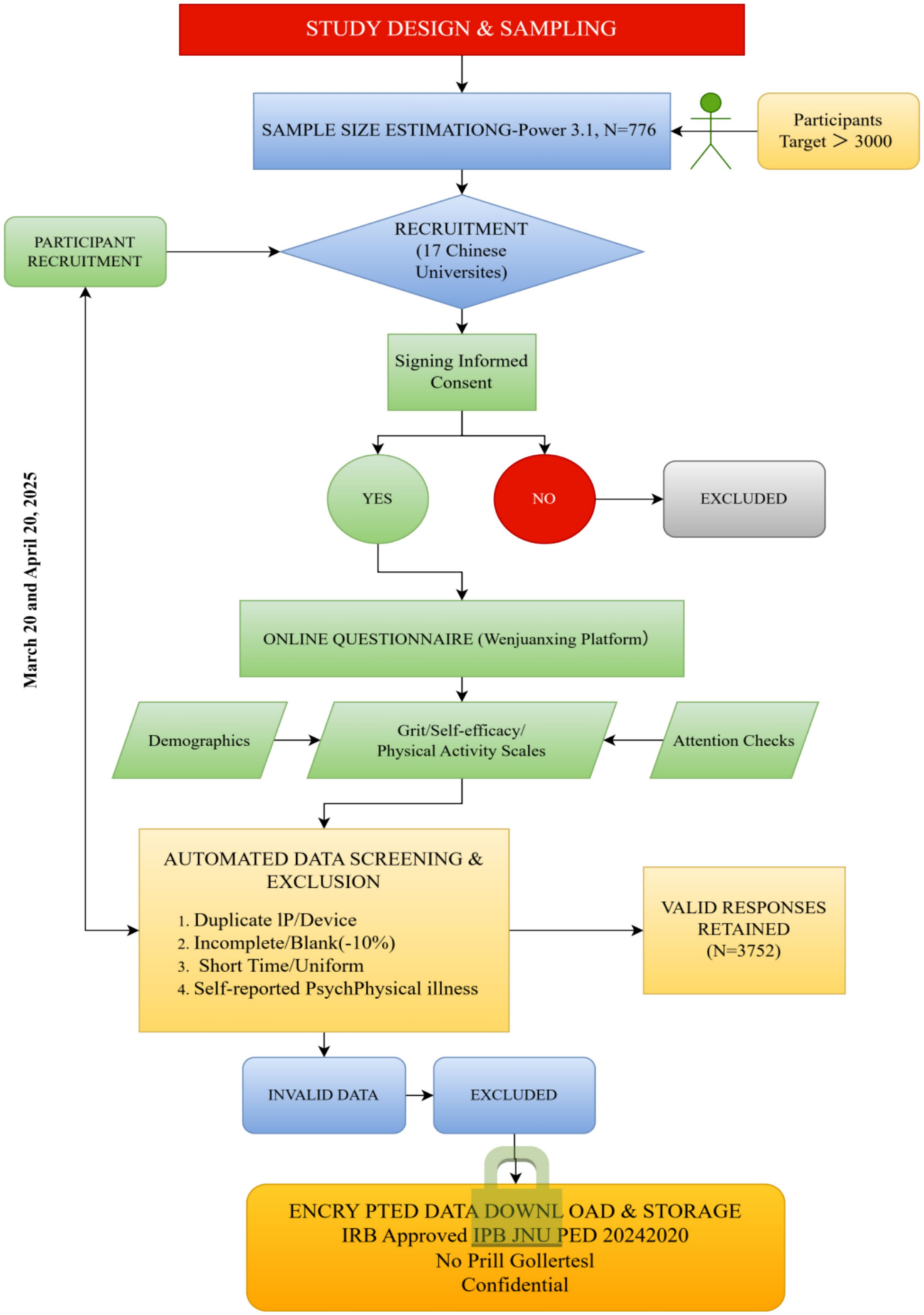
Data collection flowchart.

This study employed a cross-sectional design using convenience sampling to recruit participants from multiple universities across China (see Figure 1). Data were collected online through Wenjuanxing^1^, a reliable and secure Chinese web-based survey platform. Survey invitations containing the participation link were distributed via university counselors, student organizations, and official online groups. Students could voluntarily access the questionnaire using either computers or mobile devices.

Prior to data collection, *a priori* sample-size estimation was conducted using G*Power 3.1. The calculation was based on a multiple regression model with two predictors, assuming a small-to-medium effect size (*f*^2^ = 0.02), a significance level of *α* = 0.05, and a statistical power of 0.95. The analysis indicated a minimum requirement of 776 participants to detect significant effects. Considering the study’s potential for attrition due to missing or invalid data, the target sample size was set at more than 3,000 participants.

Data were collected between March 20 and April 20, 2025, from 17 universities located in Jiangxi, Sichuan, Hunan, Guizhou, and Guangdong. Participation was voluntary and uncompensated. Students were eligible for inclusion if they met the following criteria: (1) Full-time undergraduate or graduate enrollment at a Chinese university; (2) Age of 18 years or older; (3) Ability to understand and complete the questionnaire in Chinese; (4) Willingness to provide informed consent and participate voluntarily. Exclusion criteria were applied as follows: (1) Duplicate submissions identified through an identical IP address or device information; (2) Incomplete or blank responses (missing more than 10% of items); (3) Abnormally short completion time (<180 s) or uniform responses indicating inattentive participation; (4) Self-reported history of diagnosed psychological disorders (e.g., major depression, generalized anxiety) that might substantially influence motivational or behavioral responses; (5) Presence of a known physical illness, injury, or medical condition that restricted the ability to engage in regular PA.

A total of 3,968 questionnaires were submitted. After data screening and exclusion of invalid cases based on the criteria above, 3,752 valid responses were retained for analysis, yielding an effective response rate of 94.6%. The final sample size exceeded the calculated requirement, providing sufficient statistical power for both variable-centered and person-centered analyses.

The data-collection process was standardized across all participating universities. Students accessed the survey via an online link distributed through institutional channels. Upon entering the survey webpage, they first read a detailed information statement describing the study objectives, procedures, estimated participation time, and confidentiality policies. Participation required explicit electronic consent before proceeding. The online questionnaire took approximately 10–15 min to complete. It included demographic items (age, gender, grade level, and geographic region), followed by validated instruments measuring grit, self-efficacy, and PA. To ensure response quality, attention-check items were embedded to detect careless answering. Each IP address and device could submit only once, and the platform automatically prevented multiple entries. The dataset was subsequently downloaded in encrypted form and stored on a password-protected institutional server accessible only to the research team.

All participants completed an informed consent form electronically prior to beginning the survey. The consent form emphasized that participation was entirely voluntary, data would be kept confidential, and participants could withdraw at any time without consequence. Sensitive questions about physical or psychological conditions were included only for screening purposes and were optional. The study protocol was reviewed and approved by the Institutional Ethics Committee of the corresponding author’s university (IRB-JXNU-PEC-20240402), and all procedures complied with the ethical standards of the Declaration of Helsinki. No personally identifying information (such as name, student ID, or contact details) was collected.

### 3.2 Measures

#### 3.2.1 Grit

The present investigation utilized the Grit-S instrument for measuring grit. Initially constructed by Duckworth and Quinn (2009), this tool underwent subsequent modification by Hu to enhance its applicability for assessing persistence in athletic contexts within Chinese populations (Hu et al., 2025). The instrument features a two-dimensional structure comprising eight items, each evaluated using a five-point response format, ranging from 1 (Does not describe me at all) to 5 (Describes me perfectly). Example item: I get excited about new exercise challenges and work hard to overcome them. Within our sample, the measure exhibited robust internal consistency, yielding a Cronbach’s alpha value of 0.821, thereby confirming its psychometric adequacy.

#### 3.2.2 Physical activity

In this investigation, PA was measured using the PARS instrument, which underwent cultural adaptation by Liang (1994). This measurement tool examines three core components of PA: exercise intensity, time spent in activity, and occurrence frequency, employing a 5-point rating system for each component. Prior validation studies conducted by Yuan have confirmed the psychometric properties of this tool among Chinese participants (Yuqing et al., 2025). Physical activity was quantified by multiplying exercise intensity by adjusted duration (Duration – 1) and frequency, yielding a composite PA score. Higher values reflect elevated levels of PA participation. The instrument exhibited satisfactory internal reliability in our sample, yielding a Cronbach’s alpha coefficient of 0.827.

#### 3.2.3 Self-efficacy

The self-efficacy scale employed in this research was initially developed by Jerusalem in 1995 (Jerusalem and Schwarzer, 1995) and subsequently adapted by Hu et al. (2025) for use with Chinese populations, demonstrating satisfactory reliability and validity. The instrument comprises 10 items forming a unidimensional construct, with a sample item being “I am convinced that I can handle unexpected challenges during physical exercise.” A 4-point Likert scale is utilized, ranging from 1 (completely disagree) to 4 (completely agree), where elevated scores represent higher levels of self-efficacy. The scale demonstrated good internal consistency in the present study, with a Cronbach’s alpha of 0.826.

### 3.3 Data analysis

#### 3.3.1 Variable-centered approach

All variable-centered procedures were executed using SPSS version 26.0. The analytic sequence comprised several stages designed to verify measurement quality, explore preliminary associations, and test the hypothesized mediation model. First, descriptive statistics (mean, standard deviation, skewness, and kurtosis) were computed for grit, self-efficacy, and PA to evaluate data distribution characteristics and the suitability for parametric testing. No substantial deviations from normality were detected. Next, the internal consistency of each instrument was examined using Cronbach’s α. Reliability levels met established criteria, confirming that items measured their respective constructs consistently and could be used for hypothesis testing.

To identify potential covariates, group difference tests were conducted on participants’ demographic variables (e.g., gender, age, education, and place of birth). Demographic variables were compared using one-way ANOVA or independent-samples *t*-tests. Those variables demonstrating statistically significant differences in PA levels (*p* < 0.05) were subsequently included as control variables in the mediation analysis to account for their potential confounding influence.

Following this preliminary stage, Pearson correlation coefficients were calculated to examine the pairwise associations among grit, self-efficacy, and PA. These analyses provided an empirical basis for subsequent regression modeling.

Prior to mediation testing, potential multicollinearity among predictors was assessed. Variance Inflation Factor (VIF) and tolerance statistics confirmed acceptable independence among variables, ensuring model stability. The hypothesized mediational pathway was then tested using Hayes’s PROCESS macro (Model 4) (Yue et al., 2025). This approach relies on ordinary least-squares regression and provides both direct and indirect effect estimates. A bootstrapping procedure with 5,000 resamples was employed to generate bias-corrected 95% confidence intervals for the indirect effect. Control variables identified through variance or t-test analyses were entered to partial out their confounding impacts. This analytic strategy rigorously evaluated whether self-efficacy functioned as a link between grit and physical activity engagement within the student sample.

#### 3.3.2 Person-centered approach

To complement the variable-centered findings and capture individual-level heterogeneity, a person-centered analysis was conducted using Mplus version 8.3. The analysis applied LPA to identify distinct student subgroups characterized by unique constellations of grit and self-efficacy levels.

All LPA models were estimated via maximum-likelihood with robust standard errors (MLR). To maintain a parsimonious and interpretable structure, indicator means were freely estimated across profiles, their variances were constrained to equality, and inter-indicator covariances were fixed to zero. To ensure reproducible solutions and avoid local maxima, the estimation algorithm utilized 500 random initial starts and 100 final-stage optimizations.

Model adequacy was evaluated using multiple criteria: Akaike Information Criterion (AIC), Bayesian Information Criterion (BIC), and sample-size–adjusted BIC (aBIC), with lower values indicating better relative fit (Huang et al., 2025a). Entropy scores were inspected to gauge classification accuracy, and the Lo–Mendell–Rubin adjusted likelihood-ratio test (LMR) together with the Bootstrap Likelihood-Ratio Test (BLRT) were applied to test whether adding the profile significantly improved the model (Wan et al., 2025). Selection of the final model balanced statistical fit, classification precision, and theoretical interpretability.

After determining the optimal number of profiles, students’ physical activity levels were compared across latent groups using a one-way ANOVA conducted in SPSS 26.0. When omnibus effects were significant, Bonferroni-adjusted *post hoc* tests were used to identify specific pairwise group differences.

## 4 Results

### 4.1 Common method bias analysis

Given that data for all study variables (Grit, Self-efficacy, and PA) were collected via self-report questionnaires at a single time point, we assessed the potential influence of common method variance using Harman’s single-factor test. An unrotated exploratory factor analysis was conducted on all items. The results revealed that the first factor accounted for 30.695% of the total variance, falling well below the critical threshold of 40% (Yue et al., 2025). This finding suggests that the observed common method bias in this study is within the acceptable range.

### 4.2 Demographic information

The final sample comprised 3,752 university students (mean age = 19). As shown in Table 1, participants included 1,715 males (45.71%) and 2,037 females (54.29%). Most participants were undergraduates (*n* = 3,184, 84.86%), followed by smaller proportions of master’s students (*n* = 501, 13.35%) and doctoral students (*n* = 67, 1.79%). Urban and rural origins were relatively balanced, with 1,675 (44.64%) participants from urban areas and 2,077 (55.36%) from rural areas, respectively.

**Table 1:** Demographic characteristics and group differences among participants.

	*n*	proportion	Grit	PA	Self-efficacy
Gender			18.848^***^	17.249^***^	9.212^***^
Male	1715	45.71%			
Female	2037	54.29%			
Place of birth			−0.671	−1.778	−2.331^*^
City	1,675	44.64%			
Rural	2077	55.36%			
Education			3.120^*^	2.465	0.031
Bachelor	3,184	84.86%			
Master	501	13.35%			
Doctor	67	1.79%			

Unpaired *t*-test and ANOVA results (two-tailed test). ^*^p < 0.05, ^***^p < 0.001. Significance levels apply to subsequent tables unless otherwise noted.

Gender exhibited significant associations with grit (*t* = 18.848, *p* < 0.001), PA (*t* = 17.249, *p* < 0.001), and self-efficacy (*t* = 9.212, *p* < 0.001). Educational level was significantly related to grit (*F* = 3.120, *p* < 0.05), while place of birth showed a significant association only with self-efficacy (*t* = −2.331, *p* < 0.05).

### 4.3 Correlation analysis

As illustrated in Table 2, the descriptive statistics and bivariate correlations among the primary study variables are presented. Mean scores were 3.189 (SD = 0.924) for grit, 2.602 (SD = 0.672) for self-efficacy, and 34.240 (SD = 31.855) for PA. Skewness and kurtosis values for all variables fell within acceptable ranges, indicating approximately normal distributions suitable for parametric analyses. Correlation analyses revealed significant positive associations among all variables. Grit demonstrated positive correlation with self-efficacy (*r* = 0.542, *p* < 0.01) and positive correlation with PA (*r* = 0.419, *p* < 0.01). Self-efficacy was also positively correlated with PA (*r* = 0.326, *p* < 0.01). These preliminary findings support the hypothesized relationships and provide an empirical foundation for subsequent mediation and latent profile analyses.

**Table 2:** Variable statistics and relationships in grit, physical activity, and self-efficacy.

	*M*	*SD*	Skewness	Kurtosis	Grit	Self-efficacy	PA
Grit	3.189	0.924	−0.248	−0.535	1		
Self-efficacy	2.602	0.672	−0.099	−0.765	0.542**	1	
PA	34.240	31.855	0.498	−1.149	0.419**	0.326**	1

^**^p < 0.01.

### 4.4 The mediation analyses

Before testing the mediation model, multicollinearity diagnostics were conducted. The variance inflation factor (VIF = 1.415) was well below the threshold of 5, indicating acceptable independence among predictor variables and confirming model stability. Table 3 presents the regression coefficients for each pathway in the mediation model.

**Table 3:** Mediation effect regression results.

Variables	Model1		Model2		Model3	
	(Self-efficacy)		(PA)		(PA)	
	*β*	*t*	*β*	*t*	*β*	*t*
Grit	0.545^***^	37.956	0.293^***^	16.414	0.370^***^	24.195
Self-efficacy			0.142^***^	8.216		
Gender	0.025	0.858	−0.330^***^	−10.842	−0.327	−10.634
Education	−0.04	−0.541	0.005	0.064	−0.001	−0.008
Place of birth	0.064^*^	2.309	0.033	1.139	0.042	1.437
Age	0.002	0.139	0.006	0.378	0.006	0.393
*R* ^2^	0.295		0.214		0.200	
*F*	313.019^***^		170.113^***^		187.308^***^	

^***^p < 0.001.

As shown in Model 1, grit was significantly associated with self-efficacy (*β* = 0.545, *t* = 37.956, *p* < 0.001). Model 2 demonstrated that both grit (*β* = 0.293, *t* = 16.414, *p* < 0.001) and self-efficacy (*β* = 0.142, *t* = 8.216, *p* < 0.001) were independently associated with PA. In Model 3, when self-efficacy was excluded, grit alone was associated with PA (*β* = 0.370, *t* = 24.195, *p* < 0.001).

**Figure 2:**
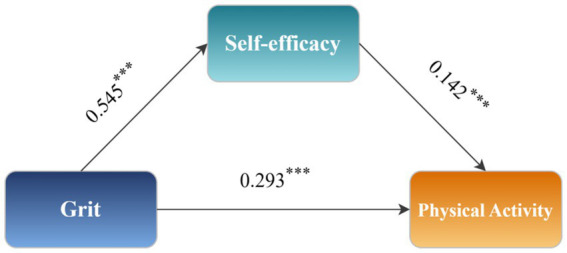
Mediation effect diagram. ***p < 0.001.

Table 4 summarizes the decomposition of total, direct, and indirect effects. The total effect of grit on PA was significant (Effect = 0.370, 95% CI [0.340, 0.400]). After controlling for self-efficacy, the direct effect remained significant (Effect = 0.293, 95% CI [0.258, 0.328]), accounting for 79% of the total effect. Critically, the indirect effect through self-efficacy was also significant (Effect = 0.077, 95% CI [0.058, 0.097]), representing 21% of the total effect. The bootstrap confidence interval excluded zero, confirming partial mediation. These findings indicate that self-efficacy serves as a significant mediator in the relationship between grit and PA, supporting Hypothesis 1 and 2. The mediation pathway is illustrated in Figure 2.

**Table 4:** Mediating effects of self-efficacy between grit and physical activity.

	Effect	Boost SE	95%CI		Percent mediated
			LLCI ULCI		
Total	0.370	0.015	0.340	0.400	
Grit → PA	0.293	0.018	0.258	0.328	79%
Grit → Self-efficacy → PA	0.077	0.010	0.058	0.097	21%

### 4.5 Latent profile analysis

LPA was performed to identify student subgroups based on their configurations of grit and self-efficacy. Models with one to five profiles were systematically compared using Mplus 8.3. Table 5 displays the fit indices.

**Table 5:** Latent profile analysis models.

Model	AIC	BIC	aBIC	LMR(*P*)	BLRT(*P*)	Entropy	Categorical probability %
Class1	17730.360	17755.280	17742.570				
Class2	16763.706	16807.316	16785.074	<0.001	<0.001	0.729	16.68%/83.32%
Class3	15311.671	15373.972	15342.197	<0.001	<0.001	0.924	16.12%/55.84%/28.04%
Class4	14911.268	14992.258	14950.951	<0.001	<0.001	0.922	10.18%/27.82%/5.68%/56.32%
Class5	14701.067	14800.748	14749.908	<0.001	<0.001	0.830	4.69%/10.13%/28.94%/26.76%/29.48%

While AIC, BIC, and aBIC decreased continuously across models, the three-profile solution demonstrated optimal entropy (0.924), indicating superior classification precision. Both LMR and BLRT tests were found to be significant for all models. Although four- and five-profile solutions showed a favorable statistical fit, they produced extremely small subgroups (4.69 and 5.68%), which limited their interpretability. Considering statistical adequacy, classification accuracy, and theoretical clarity, the three-profile model was retained as the most suitable.

**Figure 3:**
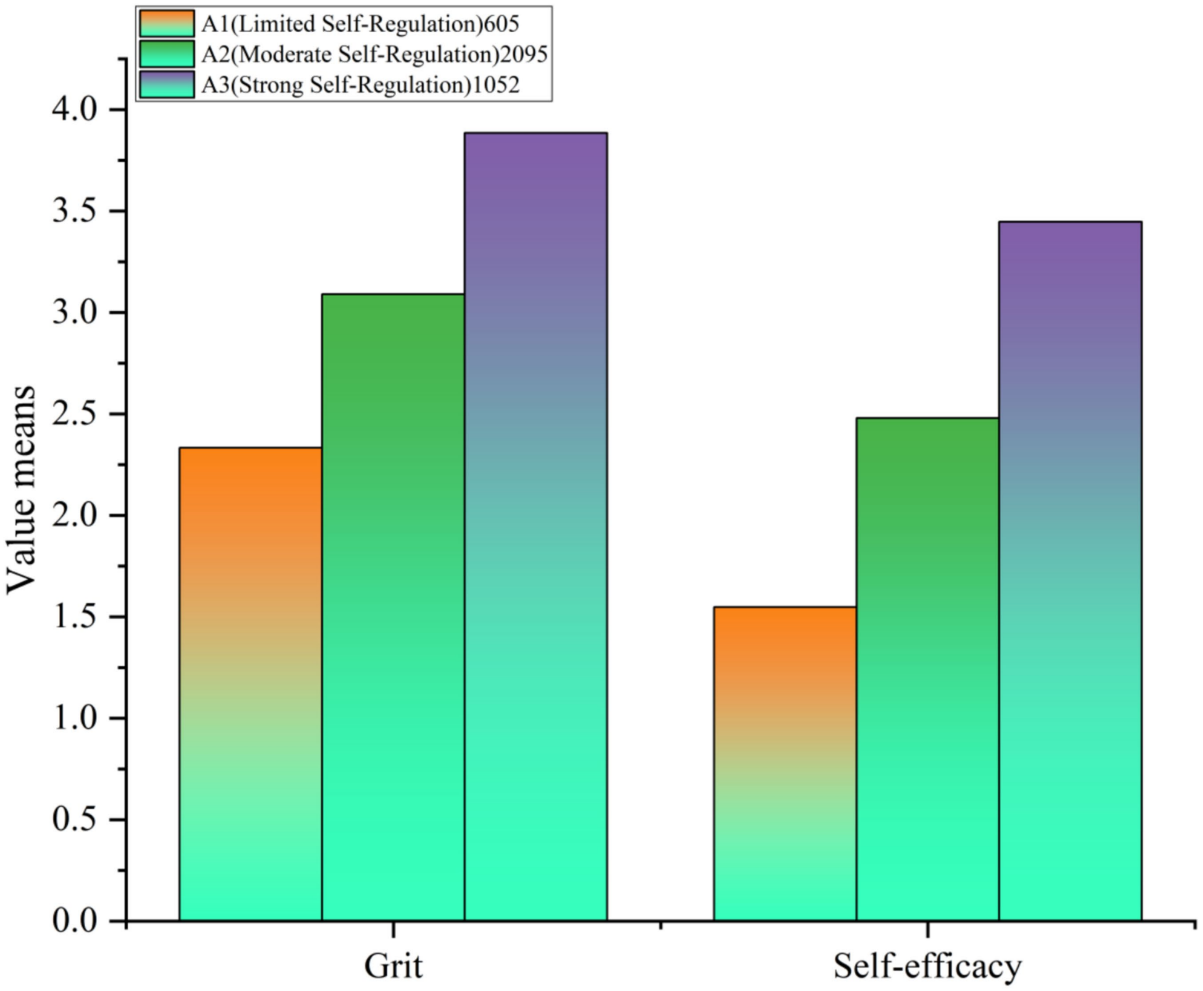
Latent profiles of grit with self-efficacy.

Figure 3 presents three distinct profiles with pronounced characteristics: Profile 1 (*n* = 605, 16.12%) exhibited low grit and self-efficacy, labeled “Limited Self-Regulation”; Profile 2 (*n* = 2,095, 55.84%) showed moderate levels on both dimensions, termed “Moderate Self-Regulation”; Profile 3 (*n* = 1,052, 28.04%) demonstrated high grit and self-efficacy, designated “Strong Self-Regulation.” These results confirm Hypothesis 3, revealing heterogeneous psychological configurations among university students.

### 4.6 Effect of latent profile classification

One-way ANOVA examined PA differences across the three profiles (see Table 6). Significant differences emerged (*F* = 225.108, *p* < 0.001, *η*^2^ = 0.107), with profile membership accounting for 10.7% of variance in PA. As shown in Table 6, the Strong Self-Regulation profile reported the highest activity levels (*M* = 49.031, SD = 29.587), followed by Moderate Self-Regulation (*M* = 31.555, SD = 31.459), and Limited Self-Regulation (*M* = 17.835, SD = 25.922).

**Table 6:** Group differences across latent profiles.

Variable	A1(605)	A2(2095)	A3(1052)	*η* ^2^	*F*
PA	17.835 ± 25.922	31.555 ± 31.459	49.031 ± 29.587	0.107	225.108^***^

A1 (Limited Self-Regulation), A2 (Moderate Self-Regulation), A3 (Strong Self-Regulation). ***p < 0.001.

Bonferroni post hoc tests revealed all pairwise differences were significant (*p* < 0.001). The pattern showed A3 > A2 > A1, confirming that distinct grit–self-efficacy configurations correspond to differential PA engagement (see Table 7). These findings support Hypothesis 4, demonstrating that distinct configurations of grit and self-efficacy are systematically associated with differential patterns of PA engagement among university students.

**Table 7:** Multiple comparisons.

Bonferroni	(I) Class	(J) Class	Mean difference (I-J)	STD. error	Sig.	95% CI		Outcome
Dependent variable						Lower bound	Upper bound	
PA	A1	A2	−13.720^*^	1.390	<0.001	−17.048	−10.392	
		A3	−31.197^*^	1.536	<0.001	−34.876	−27.517	
	A2	A1	13.720^*^	1.390	<0.001	10.392	17.048	
		A3	−17.477^*^	1.138	<0.001	−20.202	−14.752	
	A3	A1	31.197^*^	1.536	<0.001	27.517	34.875	
		A2	17.477^*^	1.138	<0.001	14.752	20.202	A3 > A2 > A1

^*^p < 0.05.

## 5 Discussion

### 5.1 The mediating role of self-efficacy

This study found that grit was positively associated with PA and that self-efficacy significantly mediated this relationship. In other words, students higher in grit tended to be more active, mainly because they felt more capable of initiating and sustaining exercise.

These findings align with prior research, which shows that grit is related to health-promoting behaviors and that self-efficacy robustly associates with exercise initiation and adherence (Hartzel et al., 2021; Liu et al., 2023). Similar mediation patterns have been reported between other positive traits (e.g., Resilience, optimism) and PA via self-efficacy, suggesting a consistent pathway across motivational constructs (Neumann et al., 2021; Jiang et al., 2025; Peng et al., 2025). Several mechanisms may explain the mediation. First, gritty students are more likely to persist through early challenges, accumulating mastery experiences that directly strengthen efficacy beliefs—Bandura’s most potent source of self-efficacy (Alhadabi and Karpinski, 2020; Kleppang et al., 2023). Second, higher efficacy reduces perceived barriers (such as time, fatigue, and setbacks), making effort more efficient and goal pursuit more autonomous (Bandura, 1982). Third, within Self-Determination Theory, the combination of sustained effort (grit) and perceived competence (self-efficacy) promotes the internalization of health goals, thereby stabilizing exercise behavior over time (Deci and Ryan, 2008).

Theoretical implications are twofold: grit provides motivational stamina, but behavior change crystallizes through efficacy-based self-regulation; thus, perseverance without perceived capability may not translate into action. Practically, interventions should pair grit-building (long-term goal framing, reflective persistence training) with efficacy-enhancing strategies (graded mastery tasks, credible peer modeling, specific feedback, and reappraisal of physiological cues). Targeting both dispositions can convert determination into durable activity habits, offering a scalable route for university health programs to increase PA participation.

### 5.2 The different latent profiles

A significant contribution of the present study is the application of a person-centered methodology, which extends beyond the population-level averages typically found in variable-centered models. In confirming Hypothesis 3, our LPA identified three statistically distinct and theoretically coherent profiles based on the interplay of grit and self-efficacy: “Strong Self-Regulation,” “Moderate Self-Regulation,” and “Limited Self-Regulation.” This finding substantiates the premise that individuals possess heterogeneous psychological configurations rather than adhering to a single motivational pathway.

The “Strong Self-Regulation” profile (Profile 3, 28.04% of the sample) represents an optimal psychological constellation for behavioral pursuit. Individuals in this group possess both high perseverance of effort (grit) and robust confidence in their capabilities (self-efficacy). This synergistic combination aligns with motivational frameworks, such as Self-Determination Theory, which posits that sustained engagement is fostered by the alignment of internal drive and perceived competence (Rigby and Ryan, 2018). High grit provides the Resilience to persist through the inherent discomforts and logistical barriers of exercise (e.g., fatigue, time constraints), while high self-efficacy provides the cognitive assurance needed to initiate action, set challenging goals, and manage setbacks effectively (Yu et al., 2024).

Conversely, the “Limited Self-Regulation” profile (Profile 1, 16.12%) characterizes a psychologically vulnerable subgroup. These students are encumbered by a dual deficit: low perseverance for long-term goals and low confidence in their ability to execute health-related actions. This combination likely creates a pernicious cycle of motivational failure (De La Cruz et al., 2021). Low self-efficacy inhibits behavioral initiation, while low grit ensures that any attempts are quickly abandoned upon facing difficulty, thus preventing the accumulation of mastery experiences necessary to build self-efficacy in the first place. This profile signifies a state of amotivation or motivational conflict that variable-centered approaches would fail to isolate.

Finally, the “Moderate Self-Regulation” profile (Profile 2) was the largest subgroup, encompassing over half of the university students sampled (55.84%). This finding is critical, as it suggests that the “average” student possesses a functional, yet suboptimal, motivational architecture. While not entirely lacking in grit or confidence, their moderate levels may render them inconsistent in their health pursuits, as they tend to succeed under favorable conditions but struggle to maintain activity when faced with significant academic or social pressures (Li et al., 2024). The identification of this large, intermediate group is paramount for public health, as they represent a primary target for broad-based interventions aimed at shifting motivational levels from adequate to optimal.

### 5.3 Effect of latent profile

Our analysis provided robust support for Hypothesis 4, demonstrating that these distinct psychological profiles are associated with significant, real-world differences in health behavior. The findings revealed a clear, hierarchical pattern in PA engagement, with the “Strong Self-Regulation” group reporting the highest levels, followed by the “Moderate” group, and finally the “Limited” group (A3 > A2 > A1).

The behavioral outcomes of the “Strong Self-Regulation” group (*M* = 49.031) align with theoretical expectations. When individuals concurrently possess both the motivational “engine” (grit) and the cognitive “steering” (self-efficacy), they appear maximally equipped to navigate the path from intention to action (Rodrigues et al., 2023). This finding empirically highlights that it is the combination of high perseverance and high confidence, rather than either trait in isolation, that is associated with the most adaptive behavioral outcomes in this sample (Datu et al., 2022).

The starkly low PA levels of the “Limited Self-Regulation” group (*M* = 17.835) identify them as a high-risk population in terms of behavioral deficits. Their minimal engagement in PA is the logical behavioral correlate of their psychological profile, which lacks both the requisite drive and perceived capability (Barbosa et al., 2024). This group exemplifies a potential failure point in health motivation, characterized by a dual deficit that corresponds to the lowest levels of health behavior.

The “Moderate Self-Regulation” profile, constituting the majority of the sample (55.84%), warrants critical theoretical attention. While this group exhibits significantly higher physical activity levels (*M* = 31.555) compared to the “Limited” profile, their engagement remains sub-optimal relative to the “Strong” profile. Theoretically, this configuration can be interpreted through Social Cognitive Theory as a state of context-dependent efficacy (Islam et al., 2023). These students likely possess sufficient self-efficacy to initiate physical tasks under normal conditions but lack the resilience, a core component of grit, required to maintain these behaviors when facing academic stressors or fatigue (Jiang et al., 2025). Unlike the “Strong” profile, their efficacy beliefs are not robust enough to serve as a buffer against external barriers (Ntoumanis et al., 2020), leading to a “competent but inconsistent” behavioral pattern (Antunes et al., 2021). This finding suggests that the primary challenge in university health promotion is not merely initiating behavior among the disengaged, but facilitating the transition of this “competent but inconsistent” majority from external regulation to autonomous persistence.

In sum, the integration of person-centered analysis provides a critical layer of granularity. While our mediation model established that self-efficacy is a key pathway linking grit to action, the LPA results reveal who within this population is most and least equipped for this pathway at this point. This dual-method approach offers a more comprehensive and granular understanding of the motivational architecture underlying PA, moving beyond a single, monolithic model to reveal distinct subgroups whose psychological configurations are differentially associated with health behavior.

### 5.4 Practical implications

The results suggest clear actions for university health initiatives. First, the finding that grit’s link to PA is channeled through self-efficacy is a key insight. It implies that programs should focus less on promoting abstract persistence and more on building tangible exercise confidence. This can be achieved through practical strategies, such as setting achievable goals or providing structured mastery experiences. Second, our profiles reveal that a uniform intervention strategy is likely to be ineffective, as students possess very different motivational makeups. Those in the “Limited Regulation” profile require foundational support for both their low confidence and persistence. The large “Moderate Regulation” group, representing the “average” student, is a prime target for broad initiatives aimed at making activity more accessible and habitual. Finally, students in the “Strong Regulation” group are not an intervention target but a potential resource; they could be engaged as peer leaders. Recognizing and responding to this student heterogeneity, rather than assuming a single pathway, is essential for improving campus-wide PA. These recommendations warrant testing in longitudinal and randomized trials.

### 5.5 Strengths and limitations

This study possesses several methodological strengths that contribute to the literature. A primary strength is the innovative adoption of a dual-method approach. By integrating a variable-centered (SEM) mediation analysis with a person-centered (LPA) approach, we were able to not only elucidate the pathway (i.e., the mediating role of self-efficacy) but also to identify the distinct profiles of individuals for whom these dynamics operate differently. This mixed-method design provides a more granular and ecologically valid understanding than either approach in isolation, moving beyond population-level averages. Furthermore, the large sample size (*N* = 3,752) lends considerable statistical power to our analyses, thereby enhancing the reliability of both the mediation model and the latent profile solution. Finally, by applying this dual framework to the under-investigated triad of grit, self-efficacy, and PA, this study offers novel insights into the heterogeneous motivational architectures within the critical population of university students.

Nevertheless, the findings must be interpreted in light of several limitations. The most significant constraint is the cross-sectional nature of the data. This design, while helpful in identifying associations, fundamentally precludes any inferences of causality or directionality. Although our mediation model is theoretically grounded (from grit to PA through self-efficacy), we cannot empirically rule out alternative or reciprocal relationships (e.g., PA engagement building self-efficacy, which in turn fosters grit).

Second, our reliance on self-report questionnaires for all variables introduces potential measurement biases. Specifically, PA data is susceptible to social desirability and Recall bias, with individuals often overestimating the duration and intensity of their activity. Future research would benefit from incorporating objective measures, such as accelerometers, to validate the PA outcomes.

Third, the generalizability of the findings is constrained. Our sample was drawn exclusively from university students in China. While this provides valuable insights into a large and important demographic, the specific motivational profiles and their prevalence may not be transferable to other populations, such as adolescents, working adults, or students in different cultural contexts. The characteristics of the identified profiles are inherently sample-dependent.

Finally, while our LPA included the core constructs of grit and self-efficacy, it is plausible that other psychological or social variables (e.g., perceived social support, barriers to exercise, or autonomous motivation) could contribute to more nuanced or different profile solutions. Future person-centered studies could incorporate a wider array of variables to capture a more comprehensive picture of students’ motivational constellations.

## 6 Conclusion

This study, adopting a dual-method approach that integrated variable-centered and person-centered analyses, offers novel insights into the relationship between grit and PA among university students. From a variable-centered perspective, our findings confirmed the critical mediating role of self-efficacy in the pathway linking grit to PA. More importantly, the person-centered Latent Profile Analysis demonstrated that students are not motivationally homogenous. Instead, they can be classified into three qualitatively distinct profiles—“Strong Self-Regulation,” “Moderate Self-Regulation,” and “Limited Self-Regulation”—which exhibited significant hierarchical differences in their PA engagement.

Taken together, our findings underscore that promoting health behaviors requires attention not only to this Grit-Self-efficacy-PA psychological pathway but also to the people (the heterogeneity of the student population). The results indicate that a combination of high grit and high self-efficacy is associated with the most adaptive health outcomes, providing an empirical basis to move beyond one-size-fits-all interventions toward more precise, personalized strategies tailored to students’ distinct motivational configurations. Crucially, the identification of the dominant “Moderate Self-Regulation” profile reveals that the decline in university physical activity is largely driven by a failure to sustain motivation among the majority, rather than a total lack of capability. Therefore, interventions should prioritize converting this “potential” group into active participants by strengthening their grit, rather than focusing solely on the most disengaged populations.

## Data Availability

The original contributions presented in the study are included in the article/supplementary material, further inquiries can be directed to the corresponding authors.

## Footnotes

1
https://www.wjx.cn/
